# Essential Oils for Bone Repair and Regeneration—Mechanisms and Applications

**DOI:** 10.3390/ma14081867

**Published:** 2021-04-09

**Authors:** Cristina Chircov, Ion Iulian Miclea, Valentina Grumezescu, Alexandru Mihai Grumezescu

**Affiliations:** 1 Faculty of Applied Chemistry and Materials Science, University Politehnica of Bucharest, RO-060042 Bucharest, Romania; cristina.chircov@yahoo.com (C.C.); micleaiulian28@yahoo.com (I.I.M.); 2 Lasers Department, National Institute for Laser, Plasma and Radiation Physics, RO-077125 Magurele, Romania; valentina_grumezescu@yahoo.com; 3 Research Institute of the University of Bucharest—ICUB, University of Bucharest, 90-92 Panduri Road, 050657 Bucharest, Romania

**Keywords:** essential oils, bone regeneration, bone repair, biocompounds

## Abstract

Although bone possesses a remarkable capacity for self-remodeling and self-healing of small defects, the continuously increasing growth of bone diseases in the elderly population is becoming a significant burden, affecting individual life quality and society. Conventional treatment options involve surgical procedures for repair and reconstruction, local debridement, autografts or allografts, bone transport, Masquelet’s two-stage reconstructions, and vascularized bone transplants. However, as such approaches often lead to disruptions of bone-regeneration processes and microbial contaminations and are often inefficient, researchers focus on developing bone-regenerative strategies and identifying novel therapeutic agents that could aid the bone-healing process. In this regard, plant-derived biocompounds, especially essential oils (EOs), have received great scientific attention in recent years, owing to their antioxidant, anti-inflammatory, and antimicrobial effects. Current studies focus on either the direct application of EOs on bone tissue or the introduction of EOs as bioactive compounds in bone scaffolds or as coatings for bone implants. Some of the EOs investigated involve St. John’s wort, rosemary, thyme, ylang, white poplar, eucalyptus, lavender, and grape seed. In this context, the present paper aims to provide an overview of the main mechanisms involved in bone repair and regeneration and the potential of EOs to address and enhance these mechanisms.

## 1. Introduction

Bone is a tissue organized into two main compartments, namely trabecular bone; i.e., cancellous or spongy; and cortical bone, i.e., dense or compact [1,2,3,4]. Structurally, bone is a nanocomposite composed of organic collagen nanofibers and inorganic compounds, such as hydroxyapatite and whitlockite, that range in size between 20 and 50 nm in the lamellar bone and 10 and 50 nm in the woven bone [5]. Through the interactions between osteocytes, osteoblasts, and osteoclasts, bone tissue is characterized by a continuously dynamic process of the new bone formation while resorting to the old tissue. Thus, bone tissue possesses a remarkable capacity for self-remodeling and self-healing when small defects occur. However, in the case of major injuries and defects, external intervention is required for restoring the functionality of bone [1,6,7].

Bone disorders, including osteoporosis, osteoarthritis, bone cancer, bone fractures, and infections, represent a great burden that affects individual life quality and society [6,8,9]. While osteoarthritis is a disease of the articular cartilage, secondary changes further affect subchondral bone remodeling, especially in the late stages [10]. With the continuously increasing growth of the elderly population, bone diseases are an important cause for direct and indirect economic losses due to severe long-term pain or physical disabilities [6,8,9]. The most common metabolic bone disorder is osteoporosis, a progressive and disabling systemic skeletal disease that is characterized by a reduced bone mass and microarchitectural deterioration of the bone, which will consequently increase bone fragility and susceptibility to fracture [8,11,12,13]. Fractures are a contributing factor for independence loss, mobility limitations, chronic pain, and increased mortality rate, and their healing is dependent upon a series of intrinsic factors, i.e., bone-loss degree, location, bone quality, fracture immobilization, blood supply, and soft tissue integrity; and extrinsic factors, i.e., age, comorbidities, smoking, medication, and nutritional status [14,15]. In this regard, the main treatment strategy involves the fixation of the broken fragments to ensure immobilization for proper healing [16,17]. Additionally, other treatment options involve surgical procedures for repair and reconstruction, local debridement, autografts or allografts, bone transport, Masquelet’s two-stage reconstructions, and vascularized bone transplants, as bone is the second-most transplanted tissue after blood [6,18,19,20]. However, such approaches often lead to disruptions of the bone-regeneration processes and microbial contaminations and are often inefficient [21].

As bone is responsible for fulfilling major functions within the body, namely support and locomotion, protection, mineral and bone matrix protein storage, and endocrine energy regulation [22], the subject of bone loss and its subsequent repair is of great importance. In this context, a clear understanding of bone loss and the underlying repair mechanisms is essential for developing successful treatment options for traumatic injuries, bone infections, metabolic bone disorders, tumors, and other associated diseases. Moreover, knowledge on this subject could further help prevent the social and economic burdens that society could face [18].

In this regard, bone-regenerative medicine has experienced considerable scientific attention in recent years [23,24]. While bone regeneration is a complex process that allows the tissue to regain and maintain its structure and function with no scar formation, certain clinical situations require an acceleration of the process. Researchers have been focusing on identifying novel therapeutic agents that could aid the bone-healing process and understand the inflammatory mechanisms that modulate this process [25,26]. Since ancient times, nature has represented a major source of bioactive compounds that exhibit therapeutic properties [27]. Plant-derived drugs are consumed by 75% of the global population and are the major form of treatment in many developing countries. As studies have shown the anti-inflammatory, antioxidant, and regenerative properties of these biocompounds, they have been widely applied in the treatment of osteoarthritis, asthma, heart diseases, hypertension, or cancer [26,28,29]. In this context, there is increasing evidence of the potential of natural biocompounds as an alternative for bone healing and regeneration, owing to their capacity for inhibiting bone resorption and tissue inflammation; increasing antioxidant defenses, tissue vascularization, and bone cell proliferation; reduced costs; and minimal side effects [26,30].

Essential oils (EOs) are secondary plant metabolites comprising complex mixtures of volatile compounds that exhibit fundamental properties for biomedical applications, including antibacterial, antiviral, antifungal, anti-inflammatory, antioxidant, analgesic, and sedative [31,32,33,34]. Furthermore, as they have shown to exhibit inhibitory effects against osteoclast activity, subsequently leading to an increase in bone mineral density [35], EOs could represent a promising candidate for developing therapeutic agents that could promote bone healing and regeneration processes. In this context, the present paper aims to provide an overview of the main mechanisms involved in bone repair and regeneration and the potential of EOs to address and enhance these mechanisms.

## 2. Bioactivities and Mechanisms of Action of Essential Oils

**Figure 1:**
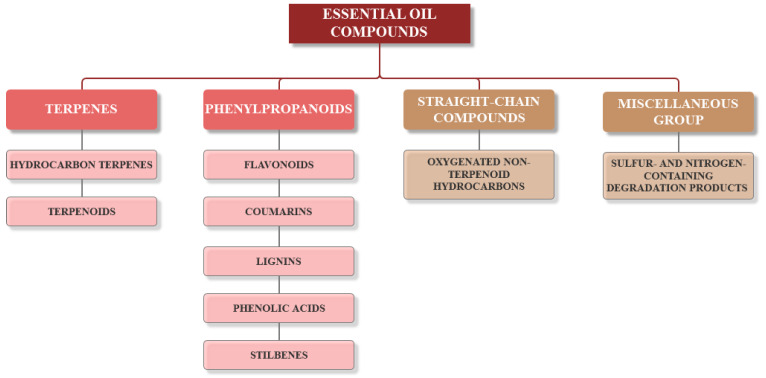
The four major classes of compounds found in essential oils.

**Figure 2:**
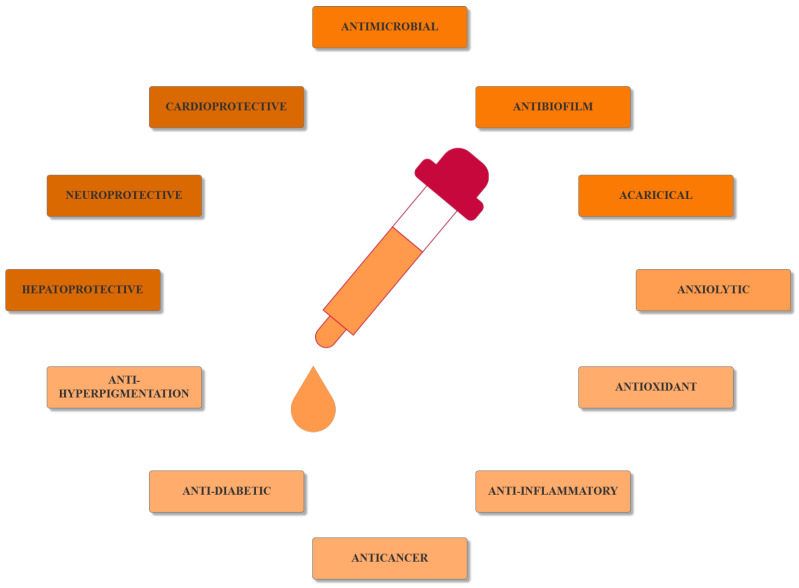
The main bioactive properties of EOs. Reprinted from an open-access source [45].

Current strategies are focusing on the use of biomaterials and biocompounds that could enhance bone-regenerative processes [36]. Therefore, natural compounds known for their anti-inflammatory, antioxidant, anticancer, and antimicrobial characters have been investigated for potential bone-healing capacities. Owing to their ability to penetrate cellular membranes and further modulate various molecular targets, from ion channels to intracellular enzymes, EOs have been extensively studied for their biological effects [37]. Although they are important sources of bioactive molecules, their effects are highly influenced by their chemical composition (Figure 1), which generally depends on the plant species, source, and part, as well as the extraction methods [38,39]. Terpenes, the most abundantly found compounds in EOs, are synthesized in the plant cell cytoplasm through the mevalonate and mevalonate-independent (deoxyxylulose phosphate) pathways [40,41]. Terpenes can be further classified based on the number of isoprene units, namely hemiterpenes (1 unit), monoterpenes (2 units), sesquiterpenes (3 units), diterpenes (4 units), sesterterpenes (5 units), triterpenes (6 units), tetraterpenes (8 units), and polyterpenes (>8 units) (Table 1) [41,42,43]. Additionally, the precise mechanisms of action are considerably difficult to understand and have not been completely elucidated, with many reports stating the synergistic effects of EO biocompounds [38]. In this context, there is a wide variety of in vitro and in vivo studies confirming the biological effects of EOs, including antibacterial, antibiofilm, antifungal, antiviral, antioxidant, anti-inflammatory, anticarcinogenic and tumor suppression, analgesic, antipyretic, anticonvulsant, hepatoprotective, cardioprotective, neuroprotective, and many more (Figure 2) [37,38,39,44]. Therefore, EOs have become a potential alternative to conventional therapy that is mostly based on synthetic compounds [39].

Reactive oxygen species (ROS) and reactive nitrogen species (RNS) are chemically reactive radical and nonradical species produced by enzymatic and nonenzymatic metabolic redox reactions involved in cellular metabolism or respiration [46,47,48,49]. On the one hand, ROS are generated through the partial reduction of oxygen to hydrogen peroxide, hydroxyl radicals, superoxide radical anions, hydroperoxyl radicals, paramagnetic singlet oxygen, ozone, or hypochlorous acid, followed by secondary reactions of these products. On the other hand, RNS, such as nitric oxide, peroxynitrite anions, or nitrogen oxide radicals, are produced through various reactions of the free radical nitrogen oxide, which is generated from arginine by the activity of nitrogen oxide synthases enzymes [47,48,49].

Generally, ROS/RNS plays fundamental roles in various physiological processes, e.g., cell signaling and signal transduction, cell-cycle regulation, phagocytosis, inflammation, enzyme and receptor activation, stressor response, gene expression, and infection prevention [47,48,50]. However, xenobiotic or environmental stressors, such as ultraviolet or ionizing radiations, pollutants, or heavy metals, significantly increase their production. In this manner, ROS/RNS reacts with macromolecules present within the cell, including proteins, lipids, or nucleic acids, consequently altering their biological functions and causing serious pathological damages to cells and tissues [46,47,48,50]. In this context, the overproduction of ROS/RNS causes oxidative or nitrosative stress, which is the global concept associated with an imbalance in the redox homeostasis due to the inability of natural antioxidant defenses to neutralize ROS/RNS and detoxify the organism of these byproducts [47,48,50,51,52].

Oxidative stress has also proved to affect bone tissues by interfering with various cellular events, ranging from cell differentiation, proliferation, and growth to cell apoptosis and disruption of the balance between osteoblastic and osteoclastic activity [53,54,55]. On the one hand, oxidative stress reduces the osteogenic potential of bone marrow mesenchymal stem cells, thus inhibiting the differentiation and proliferation of osteoblasts. Consequently, it promotes the apoptosis of mature osteoblasts, which leads to a reduction in bone formation, mineralization, and mass [53,54,55,56]. Additionally, fibronectin, a major bone extracellular matrix component involved in osteoblastic differentiation, proliferation, migration, and adhesion, is partially degraded [56]. On the other hand, as superoxide and hydrogen peroxide are involved in osteoclastic bone resorption and degradation, oxidative stress causes the upregulation of the process by enhancing the differentiation, proliferation, and activity of osteoclasts [54,56,57].

From a mechanistic point of view, oxidative stress influences these processes as they are regulated by multiple paracrine and endocrine molecules, such as ROS, cytokines, growth factors, and hormones, involved in numerous signaling pathways [54,55,58]. The main signaling systems involved in osteoblast and osteoclast activity regulation are the osteoprotegerin (OPG)/receptor activator of nuclear factor-κB (RANK)/RANK ligand (RANKL) signal-transduction pathway, the Wnt signal-transduction pathway, the mitogen-activated protein kinases (MAPK) signal-transduction pathway, through the action of extracellular signal-regulated kinases (ERK1/2), c-Jun-N terminal kinase (JNK), or p38 mitogen-activated protein kinase, transforming the growth factor-β (TGF-β) signal-transduction pathway, Notch signal-transduction pathway, and Hedgehog signal-transduction pathway [54,57,59]. These signaling pathways are further controlled by a series of interdependent transcription factors, such as canonical and noncanonical nuclear factor κB (NF-κB), c-Fos, c-Jun, Runt-related transcription factor-2 (Runx-2), Osterix (Osx), Msx1 and Msx2, and T-cell factor/lymphoid enhancer factor (TCF/LEF) [54,57,59,60].

OPG, RANK, and RANKL are molecules of the tumor necrosis factor (TNF) and its receptor superfamily [61,62], with a pivotal role in bone metabolism that was discovered at the end of the last century [63,64]. RANKL, also known as osteoprotegerin ligand (OPGL), osteoclast differentiation factor (ODF), TNF-related activation-induced cytokine (TRANCE), or TNFSF11 (TNF ligand superfamily member 11), is a homotrimeric transmembrane protein primarily expressed by osteoblasts, osteocytes, bone marrow stromal cells, and other immune cells, such as activated T cells [61,64,65]. In bone metabolism, RANKL is mainly involved in preosteoclast differentiation and osteoclast adherence, activation, and maintenance [65,66]. Moreover, the binding of RANKL with the RANK corresponding receptor, a monomeric protein expressed at the surface of osteoclast precursor cells, dendritic cells, and mature osteoclasts, will induce osteoclastogenic signals and the consequent enhancement of the osteoclast differentiation [61,62,65,66]. Subsequently, mature osteoclasts are activated and will further adhere to the surface of the bone and secrete acid and lytic enzymes, such as cathepsin K or tartrate-resistant acid phosphatase, for bone resorption [64]. Furthermore, OPG is an atypical homodimeric protein expressed by bone marrow stromal cells and osteoblasts that plays a fundamental role as an anti-osteoclastogenesis molecule [61,62,65]. Specifically, since it has been found to bind RANKL with an affinity 500-fold higher than RANK, it acts as a RANKL decoy receptor [64,65]. Therefore, OPG prevents RANKL–RANK binding and subsequently downregulates RANKL signaling, acting as a bone resorption inhibitor [64,65,66]. The OPG/RANK/RANKL system can be affected by several endogenous factors, including cytokines; i.e., TNF-a, IL-1, IL-6, IL-4, IL-11, and IL-17, hormones, such as vitamin D, estrogen, and glucocorticoids, and mesenchymal transcription factors. Additionally, OPG is also regulated through the Wnt/β-catenin signaling pathway [64].

Wnt proteins are a family of phylogenetically highly conserved secreted, cysteine-rich glycoproteins, i.e., Wnt1, Wnt2, Wnt2b, Wnt3, Wnt3a, Wnt4, Wnt5a, Wnt5b, Wnt6, Wnt7a, Wnt7b, Wnt8a, Wnt8b, Wnt9a, Wnt9b, Wnt10a, Wnt10b, Wnt11, and Wnt16; 10 seven-pass transmembrane Frizzled receptors, i.e., Fz1-10; three transmembrane tyrosine kinases, i.e., receptor-like tyrosine kinase (Ryk), tyrosine kinase-like orphan receptors (ROR), and protein tyrosine kinase 7 (PTK7); muscle-skeletal tyrosine kinase (MuSK); and two low-density lipid receptor-related protein (LRP5/6) coreceptors (Table 2) [67,68,69,70]. These proteins activate various divergent intracellular signaling pathways by receptor and coreceptor binding at the cell surface [67,68]. Specifically, there are three major pathways that have been described, namely the canonical or β-catenin-dependent Wnt/β-catenin signaling pathway, the planar cell polarity (PCP), and the Wnt/Ca2+ pathway [67,69,70]. The Wnt signaling cascade is an essential regulator of stem and progenitor cell development and maintenance during embryogenesis and adult tissue homeostasis [68,69,70,71]. Furthermore, Wnt proteins are implicated in the regulation of various cellular processes, namely cell cycle, differentiation, proliferation, motility, migration, polarity, self-renewal, metabolism, and death [68,71], acting as directional growth factors that control tissue patterning, expansion, and differentiation [68,72]. Additionally, they are also important for osteoblast differentiation and chondrocyte maturation [73]. Moreover, Wnt signaling dysregulation is linked to a multitude of diseases, including cancer, cardiovascular, neurodegenerative, and metabolic disorders, fibrosis, and bone-density disorders [67,68,72].

However, the differentiation of bone marrow stem cells into osteoblasts is mainly promoted by the canonical Wnt pathway, of which β-catenin is the key component [57,74]. In the absence of the Wnt signaling, β-catenin levels are maintained low through the constant degradation by the ubiquitin-dependent multiprotein destruction complex consisting of the scaffolding proteins Axin1/Axin2 and adenomatous polyposis coli (APC); the kinases responsible for the phosphorylation of β-catenin, namely casein kinase 1 (CK1) and glycogen synthase kinase-3β (GSK-3β); and the Dishevelled (Dvl) protein [70,74,75,76]. However, the binding of the Wnt glycoprotein to the receptor complex comprising the Fz receptor and LRP coreceptor leads to the activation of the Dvl protein and consequently to the inhibition of the destruction complex. In this manner, the β-catenin accumulated in the cytoplasm translocates into the nucleus, acting as a coactivator for the TCF and LEF and eliciting the target’s specific expression osteoblast-related genes, such as Runx-2, FoxO, alkaline phosphatase, osteocalcin, and collagen type I [55,57,70,74,75]. While Wnt signaling is modulated by several secreted proteins, including the Dickkopf proteins (Dkk1–4), the secreted Frizzled-related proteins (sFRPs1–5), and the Wnt inhibitory factor 1 (Wif1), ROS also play key roles in β-catenin accumulation regulation [70,74].

Under oxidative stress, the expression of RANKL is upregulated, while OPG is downregulated. In this context, the RANKL/OPG ratio is an indicator of the bone-remodeling process, and consequently, the intensity of bone resorption [54,57,65]. Therefore, oxidative stress has been shown to cause an increase in bone-remodeling turnover and subsequently to play key roles in the development of many age-related diseases, such as osteoporosis [54,55,57]. In this context, antioxidants have proved to contribute to osteoblast differentiation and bone formation while reducing the differentiation and activity of osteoclasts, exhibiting beneficial effects in patients with bone disorders [54]. Considering the significant impact, they could bring in disease prevention, such as cancer, heart diseases, brain disorders, or immune-system decline through their beneficial physiological actions on human cells; and in the food industry, EOs have been widely investigated for their antioxidant activities [40,77,78,79].

Their antioxidative effects have been attributed to the presence of various terpenes and phenolic compounds. Terpenes, especially from the aromatic Lamiaceae family species, such as linalool, eucalyptol, citral, citronellal, isomenthone, menthone, α-terpinene, β-terpinene, and α-terpinolene, have been widely applied as additives in food supplements for preventing oxidative stress [80,81]. Among the phenolic compounds that are known for their redox properties that play fundamental roles in free-radical neutralization and peroxide decomposition, thymol and carvacrol are the most active compounds. Additionally, antioxidant activities have also been associated with specific alcohols, ethers, aldehydes, and ketones [40]. In vitro investigations of the antioxidant properties of EOs generally include direct and indirect approaches, such as 2,2-diphenyl-1-picrylhydrayl free radical, ferric reducing–antioxidant power, β-carotene bleaching assay, total phenolic content, and ABTS radical-scavenging activity [80,82].

Furthermore, many studies have shown that high levels of ROS and oxidative stress induction in chondrocytes are the major contributors to the onset and progress of osteoarthritis [83]. In this context, it has been demonstrated that ROS upregulation in the cartilage and chondrocytes could lead to increased inflammation, and conversely, as oxidative stress is considered to be both the cause and the consequence of inflammation. On the one hand, ROS has the potential to induce the transcription of inflammation-related genes through the activation of the MAPK and NF-κB pathways, which further increase the generation of IL-1 and TNF-α. Therefore, oxidative stress is associated with the molecular-signaling dysregulation commonly observed in osteoarthritis and rheumatoid arthritis. On the other hand, like IL-1 and TNF-α and other proinflammatory cytokines and chemokines are released, macrophages and T cells become activated in the synovium, which induces ROS production and consequently promotes synovitis. As such, the relation between ROS and inflammation can be translated into one enhancing the damaging potential of the other [83,84]. Therefore, the anti-inflammatory strategy for the treatment of osteoarthritis has received significant attention [83]. However, as synthetic anti-inflammatory drugs are considered toxic and expensive, there has been an increasing interest in EOs as agents for the treatment of inflammation [85]. With many studies demonstrating the protective effects of EOs against prolonged inflammation and for improving human health, they play an important role in the process of drug discovery and development [86].

Besides their biological and pharmaceutical effects on the central nervous system and antimicrobial and antitumor actions, the subclass of sesquiterpenes has initiated the search for anti-inflammatory agents among EOs [87]. Their action is not only based on the antioxidant character, but also on the interactions with signaling cascades that involve cytokines and regulatory transcription factors and the modulation of proinflammatory gene expression. Therefore, EOs could represent a novel strategy for treating inflammatory diseases, including rheumatism, allergies, or arthritis [40].

## 3. Essential Oils for Bone Repair and Regeneration

Bone is a composition of cells embedded in a mineralized extracellular matrix consisting of 65% calcium phosphates, especially calcium hydroxyapatite, which provides the compressive strength. The remaining mass comprises organic components and water. The organic matrix consists of 90% collagen type I, which provides the tensile strength of the bone; other noncollagenous proteins, including proteoglycans, which contribute to the compressive strength of the bone; and matrix proteins, such as osteocalcin, osteopontin, and osteonectin, which promote bone mineralization and formation (Table 3). Additionally, cytokines and growth factors are involved in the activation, differentiation, growth, and turnover of the bone [15,88].

The development of bone tissue occurs through two main processes, namely intramembranous and endochondral ossification. In intramembranous ossification, neural crest-derived mesenchymal cells proliferate and condense to form compact nodules as the primitive connective tissue and further differentiate into osteoblasts in order to produce flat bones [90,91]. Osteoblasts are the cells responsible for the synthesis and mineralization of the bone matrix by secreting different extracellular matrix proteins. Thus, as the osteoid consisting of collagen–proteoglycan matrix is produced, osteoblasts are gradually embedded in it, eventually transforming into osteocytes to form the calcified tissue [88,90,92]. Osteocytes are the most abundant type of cells within bone tissue, responsible for maintaining skeleton physiological functions through mechanical strains and bone-damage sensing [88]. Endochondral ossification is the process of replacing pre-existing hyaline cartilage with bone tissue. This process is characterized by the formation of ordered zones for the proliferation and differentiation of chondrocytes in the growth plate, with distinct composition and properties of the extracellular matrix [90,91]. Specifically, the extracellular matrix of the growth plate consists of collagen type II, IX, X, and XI; aggrecan; chondroitin sulfate; hyaluronic acid; matrilins; and matrix metalloproteinases. The presence of collagen type X and the collagenase matrix metalloproteinase-13 is a contributing factor for the invasion of osteoclasts, osteogenic cells, and blood cells, leading to the ossification and maturation of the bone [90]. Osteoclasts, located at the surface of the bone, are responsible for bone-tissue resorption through calcium phosphate crystal dissolution and organic matrix decomposition [88]. Bone development and growth are influenced by a series of genetic and environmental factors, such as hormonal, diet, and mechanical factors. Additionally, it varies on the bone parts; e.g., it is faster in the proximal ends than the distal ends of long bones, as it is influenced by the intrinsic bone pressure [91].

**Figure 3:**
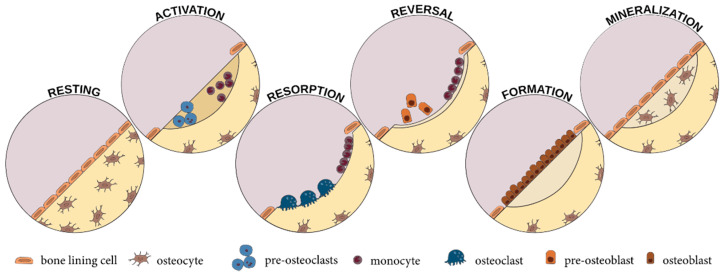
The main stages of bone remodeling [89].

Through the action of osteoclasts and osteoblasts, bone, a highly vascularized tissue, is subjected to a continuous process of remodeling that changes its internal structure in order to fulfill functional needs [88,91]. The bone-remodeling process occurs through five main sequential stages: resting, activation, resorption, reversal, bone formation, and mineralization (Figure 3). Initially, the bone surface is covered by osteoclast precursors that will subsequently be activated through physical and chemical stimuli and differentiated into mature osteoclasts to allow for bone resorption. In the reversal phase, which is a transient phase, bone resorption is inhibited, and pre-osteoblasts are recruited and subsequently differentiated to osteoblasts that will produce and mineralize the osteon. The osteoblasts undergo apoptosis or transform into lining cells or osteocytes [89,93]. The integrity of bone tissue is maintained through the delicate balance between resorption and formation processes. Variations in these processes could lead to skeletal disorders due to either excessive bone resorption, as in the case of osteoporosis, or excessive bone formation, as in the case of osteopetrosis [93].

In the cases of fractures, defects, or trauma, bone healing and regeneration is a highly organized, multipart, and reformative process that involves numerous progenitor, inflammatory, endothelial, and hematopoietic cells. Bone repair involves a cascade of biological events subdivided into three main overlapping stages: inflammation, bone production, and bone remodeling (Table 4) [88,94].

Inflammation is initiated immediately after a bone fracture, with a peak at 24 h. Bleeding into the region results in a migration of an intricate network of proinflammatory signals and growth factors, which will upregulate inflammatory molecules, including tumor necrosis factor-α, and interleukin-1, -6, -11, and -18. Subsequently, polymorphonuclear neutrophils, macrophages, and platelets are recruited and produce platelet-derived growth factor, transforming growth factor-b1, tumor-derived growth factor-β, insulin-like growth factor, and fibroblast growth factor-2 to form an initial hematoma [88,94,95].

The bone-production phases start with the substitution of the coagulated blood with a soft callus consisting of fibrous tissue and cartilage as a consequence of bone morphogenetic protein and tumor-derived growth factor-β2 and -β3 signaling. At the end of this stage, the cartilage is matured into a hard callus, which will be replaced by the woven bone [88,94,95].

The repair process’s ultimate phase is bone remodeling, which continues for more than a few months. In this step, osteoprogenitor cells differentiate into osteoblasts and osteoclasts, which will modulate the substitution of the woven bone with lamellar bone, through the signaling of interleukin-1, -6, -11, and -12; tumor necrosis factor-α; and interferon-γ. Thus, with the progressive blood circulation, the geometry and function of the damaged bone is restored [88,94,95].

As the process of bone regeneration comprises a variety of mechanisms, a complete understanding of the process requires a thorough assessment of how the individual events interact within and across multiple length and time scales [25]. Accounting for most of the functional disabilities and esthetic and psychological trauma for patients, the regeneration of bone defects caused by fractures, trauma, metabolic and congenital disorders, tumors, infectious diseases, or abnormal bone development remains one of the most important challenges in the field. In this manner, current strategies are focusing on the use of biomaterials and biocompounds that could enhance the regenerative processes [36].

Therefore, natural compounds known for their anti-inflammatory, antioxidant, anticancer, and antimicrobial characters have been investigated for potential bone-healing capacities. In this context, EOs could become ideal candidates for this purpose. However, the results described in the literature are still limited [26], and further research is required for assessing their potential in orthopedic applications. Current studies involve the use of EOs for enhancing the regeneration of bone defects and preventing or treating osteoporosis and osteoarthritis and as bioactive compounds in bone scaffolds or implants for regenerative or antimicrobial purposes.

Damlar et al. investigated the bone-healing effects of bovine-derived xenografts and *Hypericum perforatum* EO in calvaris bone bicortical defects of New Zealand rabbits. Comparative results between xenografts alone and xenografts with EOs showed improved results for the EO models, with reduced residual grafts and enhanced de novo bone formation [96].

Furthermore, Kania et al. studied the effects of *Cinnamomum burmanini* Blume EOs in ovariectomized Wistar rats on bone-turnover markers, mineral elements, and mesostructure. Specifically, administration at 12.5, 25, and 50 mg/kg body weight doses resulted in an attenuated increase of serum C-telopeptide collagen type I and osteocalcin, thus proving the EO’s potential to normalize bone-turnover markers and achieve the mesostructure of hydroxyapatite crystal growth [97]. Additionally, Elbahnasawy et al. evaluated the protective effects of *Thymus vulgaris* and *Rosmarinus officinalis* EOs against osteoporosis in male Sprague–Dawley rats with low calcium intake. EO administration resulted in bone-loss inhibition, plasma calcium and vitamin D3 increases, bone mineral-density improvement, and inflammation and oxidative stress prevention, which proves their efficiency for counteracting bone resorption and osteoporosis [98]. Moreover, Sapkota et al. evaluated the effects of thymol, the key compound contributing to thyme leaves’ aroma, on receptor activator NF-κB ligand-induced osteoclastogenesis in murine macrophage RAW264.7 cells and bone-marrow-derived macrophage cells and lipopolysaccharide-induced bone loss in ICR mice. Thymol significantly reduced the formation and differentiation of osteoclasts without cytotoxic effects and lipopolysaccharide-induced bone loss, thus proving its therapeutic potential for metabolic bone disorders [99].

Similarly, the effects of EOs on preventing or ameliorating osteoarthritis have been widely investigated. In this context, Belkhodja et al. performed radiographic and histologic evaluations of the effects of *Rosmarinus officinalis* and *Populus alba* on Wistar rat models of knee osteoarthritis. Results showed significant decreases in the Mankin scores for EO-treated mice compared to the untreated group, with a slightly higher difference for the *Populus alba* group [100]. Furthermore, Funk et al. investigated the anti-inflammatory activity of ginger EOs in female Lewis rats with streptococcal cell wall-induced arthritis. Administration of the EO prevented chronic joint inflammation, and results suggested that the anti-inflammatory effects could be attributed to the synergistic effects of its biocompounds [101]. Moreover, Bi et al. investigated the effects of *Notopterygium incisum* volatile EOs against the production of nitric oxide in RAW264.7 cells, which decreased by more than 50%. Additionally, the EO ameliorated adjuvant-induced arthritis in rat models in a dose-dependent manner and inhibited EAhy926 cell proliferation. Therefore, the antioxidant, anti-inflammatory, antiproliferative, and antiangiogenic effects make *Notopterygium incisum* EOs potential candidates in the treatment of osteoarthritis [102]. α-bisabolol, a sesquiterpene found in various EOs, such as chamomile, has been studied for its anti-inflammatory and chondroprotective effects. Its administration on human chondrocytes treated with advanced glycation end products to mimic osteoarthritis suppressed inflammation and extracellular matrix degeneration by blocking nuclear factor kappa B, p38, and c-Jun N-terminal kinase signaling. Additionally, α-bisabolol ameliorated radiological and histopathological changes in mouse models, proving its potential for osteoarthritis therapy [103]. Similar results were obtained by Gomes et al., who applied myrtenol, a bicyclic monoterpene with anti-inflammatory properties, on rat models of chronic osteoarthritis [104]. Additionally, studies evaluating the effects of aromatherapy massages in patients with osteoarthritis using sweet almond, apricot kernel, lavender, eucalyptus, and ginger EOs proved their potential in ameliorating knee pain, which could be applied as a routine adjuvant therapy [105,106].

**Figure 4:**
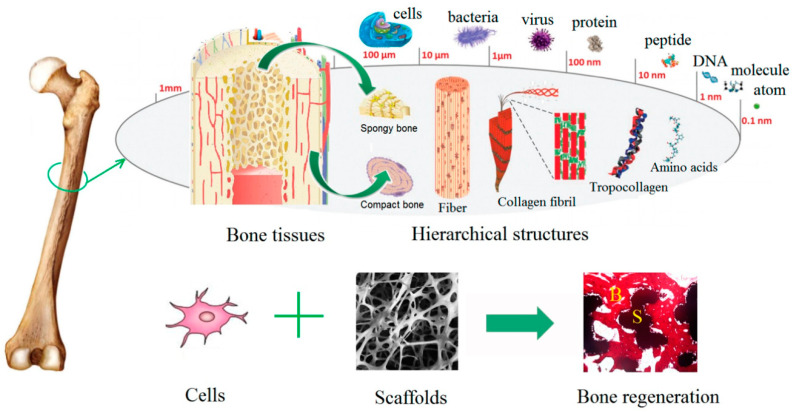
Schematic representation of bone structure hierarchy and anatomy and the synergistic effects of cells and scaffolds involved in bone regeneration. Reprinted from an open-access source [114].

In addition, EOs could be applied as bioactive compounds in scaffolds designed for bone-regeneration purposes (Figure 4). In this context, electrospun polyurethane scaffolds treated with EOs and metallic nanoparticles have been widely investigated. Chao et al. treated polyurethane scaffolds with grape seed EOs and honey and propolis. Results showed that neither of the scaffolds exhibited cytotoxic effects on red blood cells and human fibroblast cells, thus proving blood compatibility and cell viability rates. Additionally, the addition of the biocompounds increased hydrophilicity and thermal stability and reduced surface roughness [107]. Mani et al. treated polyurethane nanofibers with ylang EOs and zinc nitrate, which improved thermal stability and mechanical properties and reduced surface roughness. Microbiology tests revealed improved biocompatibility and anticoagulant properties and increased calcium deposition for the treated samples [108]. Similar results were obtained by Jaganathan et al. by treating polyurethane scaffolds with rosemary EOs and copper sulfate, which exhibited antibacterial, bone mineralization, and osteoblast cell adhesion and proliferation activities [109]. Zhang et al. treated electrospun polyurethane with lavender EOs and cobalt nitrate, and results proved delayed blood coagulation, nontoxic behavior on fibroblast cells, and bone mineralization properties [110]. Moreover, Banerjee et al. developed porous fluorescent nanocrystalline erbium-doped hydroxyapatite treated with eucalyptus, frankincense, tea tree, and wintergreen EOs. The obtained nanocomposites exhibited moderate biocompatibility toward WI-38 cells, antimicrobial activities against *Escherichia coli* and *Staphylococcus aureus*, cytotoxic effects against breast cancer cell line MDA-MB 468, and pH-dependent EO release profiles [111]. Florea et al. also demonstrated the potential of eucalyptus EOs in collagen type I and hydroxyapatite composite scaffolds to promote bone regeneration and inhibit antimicrobial activities [112]. Moreover, Polo et al. combined the regenerative effects of the commercially available Surgibone calcium phosphate microparticles and vanillin’s antimicrobial effects. Results showed antibacterial effects toward *Escherichia coli* and biocompatibility on MG-63 human osteoblast-like cells, but further in vivo studies are required to confirm these biomaterials’ efficacy [113].

Additionally, EOs could also be applied for coating metallic implants in order to prevent microbial contamination and infections. In this regard, treatment of Ti6Al4V with peppermint EOs [115] and farnesol [116] could represent a promising coating strategy that prevents bacterial adhesion and biofilm development.

## 4. Conclusions and Future Perspectives

Bone healing and regeneration is a highly organized, multipart, and reformative process that involves numerous progenitor, inflammatory, endothelial, and hematopoietic cells and a cascade of biological events. As the intrinsic capacity of self-remodeling and self-healing is insufficient in the case of bone fractures, defects, or trauma, additional treatment is required. As conventional options are associated with a variety of drawbacks and are relatively ineffective, innovative strategies are fundamental. EOs, which have long proven their antimicrobial, antioxidant, and anti-inflammatory effects, could be efficiently used for bone repair and regenerative applications. In this context, studies focus on applying EOs for enhancing the regeneration of bone defects and preventing or treating osteoporosis and osteoarthritis and as bioactive compounds in bone scaffolds or implants for regenerative or antimicrobial purposes. The most commonly used EOs include St. John wort, cinnamon, thyme, rosemary, white poplar, ginger, and *Notopterygium* root EOs, which have proven to improve bone properties, such as mineral turnover marker normalization, bone-loss inhibition, plasma calcium and vitamin D3 increases, bone mineral-density improvement, and inflammation and oxidative stress prevention Furthermore, grape seed, ylang, rosemary, eucalyptus, frankincense, tea tree, and wintergreen EOs applied in scaffolds have improved biocompatibility and bone regeneration capacities, while also preventing microbial colonization. However, the publications available in the literature are still limited, with most studies only focusing on animal experiments. Thereby, further studies are required in order to confirm the efficiency of these biocompounds. Additionally, assessing the precise chemical composition and the underlying mechanisms of action is necessary for a thorough understanding of the field.

## Figures and Tables

**Table 1:** Summary of the main hydrocarbon terpenes and the associated number of isoprene units and carbon atoms.

Compound	Isoprene Units	Carbon Atoms	Examples and Chemical Structures					
Hemiterpenes	1	5	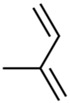					
			isoprene					
Monoterpenes	2	10	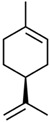	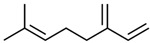		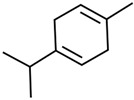		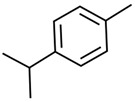
			limonene	myrcene		γ-terpinene		p-cymene
			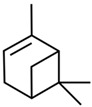		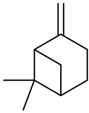		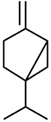	
			α-pinene		β-pinene		sabinene	
Sesquiterpenes	3	15	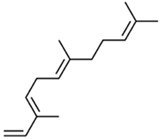		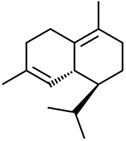		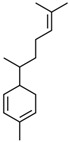	
			α-farnesene		δ-cadinene		zingiberene	
			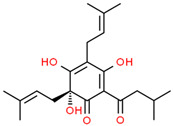	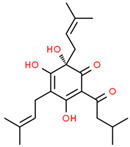		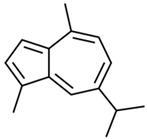		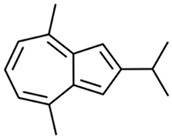
			R-humulone	S-humulone		guaiazulene		elamazulene
Diterpenes	4	20	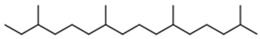		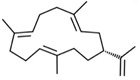		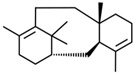	
			phytane		cembrene A		taxadiene	
			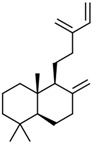		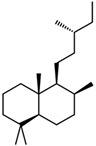		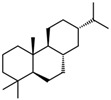	
			sclarene		labdane		abietane	
Sesterterpenes	5	25	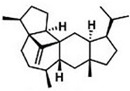		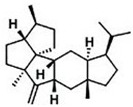		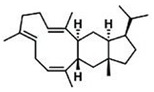	
			astellatene		boleracene		caprutriene	
			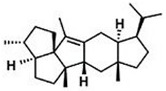		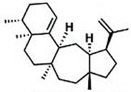		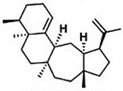	
			retigeranin B		brarapadiene A		brarapadiene B	
Triterpenes	6	30	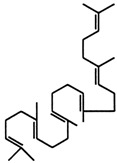		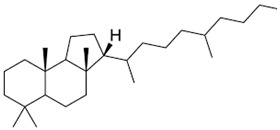		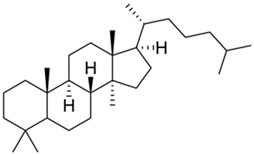	
			squalene		malabaricane		lanostane	
			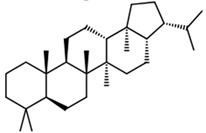		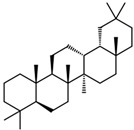		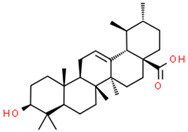	
			hopane		oleanane		ursolic acid	
Tetraterpenes	8	40	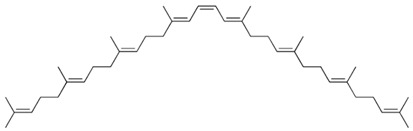			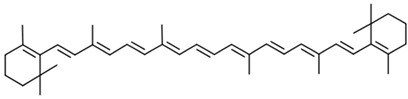		
			phytoene			β-carotene		
			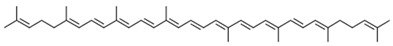			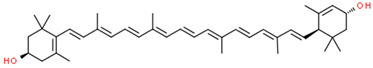		
			lycopene			lutein		
			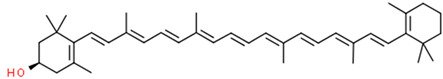					
			cryptoxanthin					

**Table 2:** Summary of the Wnt protein family members.

Protein Class	Members
Secreted cysteine-rich glycoproteins	Wnt1, Wnt2, Wnt2b, Wnt3, Wnt3a, Wnt4, Wnt5a, Wnt5b, Wnt6, Wnt7a, Wnt7b, Wnt8a, Wnt8b, Wnt9a, Wnt9b, Wnt10a, Wnt10b, Wnt11, and Wnt16
Seven-pass transmembrane Frizzled receptors	Fz1, Fz-2, Fz-3, Fz-4, Fz-5, Fz-6, Fz-7, Fz-8, Fz-9, and Fz-10
Transmembrane tyrosine kinases	Ryk, ROR, and PTK7
Muscle skeletal tyrosine kinase	MuSK
Low-density lipid receptor-related proteins co-receptors	LRP5 and LRP6

**Table 3:** The main components of bone organic extracellular matrix [89].

Collagens	Proteoglycans/Glycosaminoglycans	Matrix Proteins
Collagen type I Collagen type III	Decorin Lumican Biglycan Epiphycan Keratocan	Osteocalcin Osteopontin Osteonectin Sialoprotein

**Table 4:** The key events and the signaling molecules involved in the bone repair and regeneration process [18,88,94,95].

Bone Repair Phase	Key Events	Signaling Molecules
Inflammation	Production of pro-inflammatory cytokines, chemokines, and growth factors Recruitment of polymorphonuclear neutrophils, macrophages, and platelets Activation of the blood coagulation cascade Formation of hematoma Angiogenesis	Tumor necrosis factor-α Interleukin-1, -6, -11, -18 Platelet-derived growth factor Transforming growth factor-b1 Tumor-derived growth factor-β Insulin-like growth factor Fibroblast growth factor-2
Bone production	Differentiation of progenitor cells into chondrocytes Formation of fibrocartilage Fibrocartilage calcification Woven bone deposition	Bone morphogenetic protein Tumor-derived growth factor-β2 and -β3
Bone remodeling	Differentiation of osteoprogenitor cells into osteoblasts and osteoclasts Resorption of woven bone Deposition of lamellar bone	Interleukin-1, -6, -11, and -12 Tumor necrosis factor-α Interferon-γ

## Data Availability

Not applicable.
